# Social Distancing Compliance under COVID-19 Pandemic and Mental Health Impacts: A Population-Based Study

**DOI:** 10.3390/ijerph17186692

**Published:** 2020-09-14

**Authors:** Sheng Zhi Zhao, Janet Yuen Ha Wong, Yongda Wu, Edmond Pui Hang Choi, Man Ping Wang, Tai Hing Lam

**Affiliations:** 1 School of Nursing, LKS Faculty of Medicine, The University of Hong Kong, Pokfulam, Hong Kong, China; lubabezz@hku.hk (S.Z.Z.); janetyh@hku.hk (J.Y.H.W.); yongdang@connect.hku.hk (Y.W.); ephchoi@hku.hk (E.P.H.C.); 2 School of Public Health, LKS Faculty of Medicine, The University of Hong Kong, Pokfulam, Hong Kong, China; hrmrlth@hku.hk

**Keywords:** COVID-19, social distancing, compliance, stay-at-home, mental health, public health intervention

## Abstract

The success of public health measures for controlling the coronavirus disease 2019 (COVID-19) pandemic relies on population compliance. We analyzed compliance with social distancing and its associations with mental health. The Hong Kong COVID-19 Health Information Survey was conducted from 9–23 April 2020 on 1501 adults randomly sampled for landline telephone interviews (*n* = 500) and online surveys (*n* = 1001). Compliance with social distancing and staying-at-home, stress (Perceived Stress Scale-4), anxiety (General Anxiety Disorders-2), and depressive symptoms (Patient Health Questionnaire-2) were collected. The associations between mental health symptoms and compliance were examined by multivariable regression models. Of the 1501 respondents (52.5% female, 72.3% aged 18–59 years), 74.2%, 72.7%, and 59.7% reported avoiding going out, going to crowded places, and attending social gatherings of more than four people, respectively. Most respondents had stayed-at-home for at least four of the past seven days (58.4%; mean 4.12, Standard Deviation 2.05). Adoption, perceived effectiveness, and perceived compliance with social distancing were associated with lower stress levels and less anxiety and depressive symptoms (all *p* < 0.01). However, more days stayed-at-home were associated with more depressive symptoms (adjusted Odds Ratio 1.09; 95%Confidence Interval 1.00, 1.18). The long-term psychological impact in relation to social distancing and staying-at-home requires further investigation.

## 1. Introduction

The coronavirus pandemic 2019 (COVID-19) has caused millions of confirmed cases and thousands of deaths since early 2020. Driven by the increasingly frequent worldwide transportation of those with asymptomatic patients, the infection is spreading more rapidly and widely than ever [1]. Public health measures such as border restrictions, bans on public gatherings, closures of schools and nonessential businesses, stay-at-home and social distancing have been enforced in many countries. At the early stage of the epidemic, Hong Kong responded quickly with precautionary behaviors including mask wearing and hand hygiene [2,3,4]. Solidarity and altruism were aroused for collective community protection [5]. Isolation, quarantine, contact tracing and social distancing were the main measures to prevent community outbreak and were effective in that the transmission of COVID-19 remained low (daily effective reproduction number R_1_ approximately equal to 1) by early March without lock down [2]. With a long incubation period (up to 14 days), substantial pre-symptomatic transmission and globally imported cases, these public health measures were needed for a long period of time [6,7]. Engagement and compliance with these measures, especially social distancing, in mitigating silent transmission was decisive to the efficacy of local outbreak containment.

The prevalence of anxiety and depression have increased during the COVID-19 pandemic [8]. Psychological distress related to the pandemic is well documented [9,10]. Self-isolation and stay-at-home could result in reduced social interaction, unintentional changes in daily routine and sleep disturbances, and lead to mental distress symptoms [11]. Increased levels of stress, depression, confusion, loneliness and anxiety were associated with quarantine measures [11,12]. Mental health responses to the public health measures have concerned policy makers and healthcare providers in order to balance pandemic control and other damage control. Compliance with stringent social distancing measures may increase psychological distress [13,14] as echoed by previous studies of the SARS pandemic which found that compliance with quarantine had increased emotional distress [15]. However, it is uncertain how compliance with social distancing in this highly transmissive pandemic, where infection control mainly relies on non-pharmaceutical interventions [2] and social distancing, will affect mental health, as these measures will substantially change the population’s behavioral patterns and daily functioning, but contribute to changes in community health [16,17,18].

Hong Kong had its first confirmed case on 23 January 2020 [19]. Public health measures were effective as shown by only a few cases per day for the first eight weeks and were well managed by the healthcare system with few deaths [2,20]. However, the large influx of Hong Kong residents from the UK and other countries in late March led to sudden increases of imported cases which then led to clusters of local cases [19]. On 29 March, during the peak of the outbreak, bans on gatherings of more than four people in public places, closure of leisure venues and restrictions on restaurant capacity were issued and social distancing was further enhanced. Up to 23April 1035 cases were reported. During this pandemic, health communication was transparent as facilitated by social media and other online platforms with timely case reports, contact tracing, and personal protection measures. Without an entire lockdown, public health interventions allowed a certain freedom for voluntary compliance. We aim to assess this compliance with social distancing and stay-at-home measures, and to examine the associations of these measures with mental health symptoms (i.e., stress level, anxiety, and depressive symptoms).

## 2. Materials and Methods

### 2.1. Study Design and Sampling

The Hong Kong COVID-19 Health Information Survey (CoVHInS) was a population-based dual sampling landline and online survey conducted from 9–23 April 2020. The target population was general Hong Kong residents aged 18 or above. Social Policy Research Limited, a reputable local survey agency, was commissioned to conduct the survey.

We adopted two-stage random sampling in the landline survey using the Web-based Computer-Assisted Telephone Interview system (Web-CATI). Residential telephone directories that covered approximately 76% of Hong Kong residents [21,22] were used to generate a random list of telephone numbers for interview. Invalid numbers (e.g., fax line, non-residence line and non-working line), nonresponses, and ineligible households were excluded. After telephone contact had been successfully established with a target household, one eligible person was selected using the “next birthday” rule (i.e., the household member whose birthday was nearest to the interview date was selected). All telephone interviewers completed a half-day training related to COVID-19 knowledge, contents of the questionnaire, sampling procedures and interviewing techniques. Briefing and de-briefing sessions were arranged during data collection, and rigorous quality checks were adopted (17.2% were checked) to ensure research fidelity. Each interview took approximately 25 min. Among the 816 valid telephone number sampled (305 refused, 11 dropout), 500 respondents completed the interview yielding a response rate of 61.3%.

The online survey was conducted on a representative panel of Hong Kong residents. This panel was previously formed by inviting local mobile phone users to join. All mobile phone numbers (prefix starting with 5, 6 or 9), which covered over 90% of Hong Kong residents, received an invitation message. These numbers were generated using the Numbering Plan for Telecommunication Services in Hong Kong provided by the Office of the Communications Authority (OFCA). A total of 100,079 residents covering diverse socio-economic backgrounds joined the panel. Stratified random sampling by sex and age was adopted to select a random list of panel participants, who were then invited to join the online survey by an invitation text message. Participants self-administered the questionnaire via Web-CATI. The response rate was 61.7% (1001 of 1623 eligible panel participants).

Ethics approval was granted by the Institutional Review Board (IRB) of the University of Hong Kong/Hospital Authority Hong Kong West Cluster (UW 20-238). Informed consent was obtained from all respondents before answering the questions.

### 2.2. Measurements

The adoption of social distancing measures (yes/no) since the first case confirmed in Hong Kong included: (i) avoid going out, (ii) avoid going to crowded places, (iii) avoid going to high-risk places (e.g., bar, wet market, hospital), (iv) avoid social gatherings of more than four people (government regulation, with penalty, issued on 29th March), (v) avoid greetings with physical contact such as handshaking, hugging and kissing, and (vi) keep 1.5 meters from others in public. The total number of social distancing measures was calculated (ranged 0–6). Perceived overall effectiveness of and perceived compliance with the above social distancing measures were measured on a scale ranging from 0–10 with higher scores indicating higher perceived effectiveness and compliance. Stay-at-home, which was voluntary, was measured by the number of days at home except for essential tasks in the past seven days. Personal protection measures including: (i) wearing surgical mask, (ii) washing hands with alcohol-based sanitizers, (iii) using alcohol to clean daily necessities, and (iv) adding water to household drainage system in the past seven days were recorded.

Stress level in the preceding month (from early-March to mid-April 2020) was assessed by the four-item Perceived Stress Scale (PSS-4) [23]. PSS-4 consists of four items measuring the degree of ability to cope with existing stressors (positive elements, two items, Cronbach’s α = 0.68) and the degree of lack of control and affective reactions (negative elements, two items, Cronbach’s α = 0.69). Each item is rated on a Likert-like scale ranged from 0 (never) to 4 (very often). Higher total scores on the four items (0–16) indicate a higher perceived stress level. The Chinese version of PSS-4 has been validated in our previous study with satisfactory internal consistency (Cronbach’s α = 0.67) [24].

Anxiety and depressive symptoms in the past two weeks (from late-March to mid-April 2020) were assessed using the four-item Patient Health Questionnaire (PHQ-4). PHQ-4 consists of the two-item General Anxiety Disorder (GAD-2) and the two-item Patient Health Questionnaire (PHQ-2) [25,26]. GAD-2 covered two core criteria for generalized anxiety that screen for social panic and anxiety disorders. PHQ-2 measured depressive symptoms, depressed mood and loss of interest, two core diagnostic criteria for major depression disorder [26]. Each item scores on a Likert-like scale ranging from 0 (not at all) to 3 (nearly every day). Subscales of the GAD-2 and PHQ-2 scores range from 0–6 with a score of ≥3 indicating anxiety and depression symptoms [27,28]. We have previously validated the Chinese version of the PHQ-2 in the Hong Kong population [29]. The internal consistency of GAD-2 (Cronbach’s α = 0.81) and PHQ-2 (Cronbach’s α = 0.81) were positive in this study.

Sex, age, marital status (never married, married or cohabited, divorced or separated, or widowed), current living arrangement (living alone, co-living with family members, or co-living with other people), and socioeconomic status (SES) including educational attainment (primary or below, secondary, or tertiary), employment status (full-time work, part-time work, student, housemaker, unemployed or retired) and monthly personal income (HK$ ≤ 10,000; 10,001–20,000; 20,001–30,000, 30,001–40,000, or ≥40,001; US$1 = HK$7.8) of the respondents were recorded.

### 2.3. Statistical Analysis

We used Chi-squared test and *t*-test to compare sociodemographic characteristics and mental health symptoms between the landline telephone and online self-administrated samples. To improve the representativeness of the sample, all data were weighted according to provisional figures obtained from the Census and Statistics Department on the sex, age, and education attainment distributions of Hong Kong’s general adult population in 2016. Multivariable linear regression was used to examine the associations with sociodemographic characteristics of number of social distancing measures adopted, number of days stayed-at-home and perceived compliance with social distancing measures. The association of mental health symptoms including stress, anxiety and depression with the number of measures adopted, number of days stayed-at-home and perceived effectiveness and compliance were calculated by multivariable linear (for stress) and logistic (for anxiety and depression) regressions. In regression model 1, potential sociodemographic confounders including sex, age, educational attainment, current employment status, and monthly income were adjusted. We additionally adjusted for the four personal protection measures in regression model 2. The association between personal protection measures and mental health symptoms were analyzed by multivariable regression models adjusted for sociodemographic factors and social distancing. Effect modifications by age (18–59, 65+ years) and education attainment (primary or below, secondary, and tertiary) on the associations between mental health symptoms, stay-at-home and compliance with social distancing were assessed using the interaction terms. Analyses were performed using STATA version/MP 15.1 (StataCorp LP, College Station, TX, USA).

## 3. Results

There were no significant differences in the sociodemographic characteristics of the landline telephone sample (*n* = 500) and the online self-administrated sample (*n* = 1001) (all *p* > 0.05; Table 1). In the combined weighted sample, 52.5% were female, 72.3% aged 18–59 years, 66.1% were married or cohabitated, and 92.9% were co-living with family members. Three quarter (76.8%) of respondents had a secondary or higher education, 62.9% were employed and 68.2% had a monthly household income of HK$20,000 or lower. The average stress score was 7.20 (standard deviation, SD: 2.12), and 15.8% and 14.8% of the respondents had experienced anxiety and depressive symptoms, respectively.

Table 2 shows that most respondents adopted the social distancing measures including avoid going out (74.2%), avoid going to crowded places (72.7%), avoid social gatherings of more than four people (59.7%), avoid going to high-risk places (54.8%), and avoid handshaking, hugging and kissing (50.7%), but only 42.9% kept 1.5 meters away from others. Respondents adopted on average 3.55 (SD 1.76) social distancing measures and most had stayed-at-home for at least four days in the past seven (58.4%; mean 4.12, SD 2.05). Respondents perceived the social distancing measures to be effective in containing the infection (mean 7.34, SD 1.88) and perceived a moderate level of compliance (mean 6.68, SD 1.98).

Table 3 shows that older age and higher education attainment were significantly associated with the adoption of more social distancing measures, more days stayed-at-home and perceived higher level of compliance with social distancing (all *p* for trend <0.01). Being female was associated with more days stayed-at-home (adjusted β 0.49; 95%CI 0.30, 0.68) and perceived compliance with social distancing (adjusted β 0.27; 95%CI 0.07, 0.46). Compared with respondents in full-time work, those who were economically inactive spent more days at home and reported higher compliance with social distancing (all *p* for trend <0.001). Having higher income was associated with increased perceived compliance with social distancing (*p* for trend <0.001).

Table 4 shows that adopting more social distancing measures was significantly associated with lower stress level (adjusted β −0.12; 95%CI −0.18, −0.06) and lower risk for anxiety symptoms (adjusted OR 0.87; 95%CI 0.79, 0.95) after the models were adjusted for sociodemographic and personal protection measures (model 2). Perceived effectiveness and compliance with social distancing measures were associated with lower stress levels and risks for anxiety and depressive symptoms (all *p* < 0.001). Consistently, personal protection measures, including mask wearing, use of alcohol to clean daily necessities and adding water to the household drainage system were associated with lower stress levels and lower risks for anxiety and depressive symptoms (all *p* < 0.01) (Supplementary Table S1). Nevertheless, more days stayed-at-home was significantly associated with increased risk for depressive symptoms (adjusted odd ratio (OR) 1.09; 95%CI 1.00, 1.18). Supplementary Table S2 shows that more days stayed-at-home was associated with anxiety (*p* for interaction = 0.005) and depressive (*p* for interaction = 0.008) symptoms especially among respondents with older age (60+ years). More days stayed-at-home was associated with a higher stress level especially among respondents with primary or lower education attainment (*p* for interaction = 0.014).

## 4. Discussion

We have provided the first evidence on compliance with non-pharmaceutical community containment strategies including stay-at-home and social distancing and their associations with mental health symptoms during the COVID-19 pandemic. Random samples from landline and mobile phone increased the representativeness of the study. The effectiveness of public health interventions depends on the degree of population engagement and compliance in practice. Avoiding going out and avoiding social gatherings were mostly practiced, which may have resulted in the achievement of suppressing the first wave of community outbreak in February and March (first eight weeks) and in mitigating the second wave, due to imported cases, in April and May [2]. Most respondents have voluntarily stayed at home for at least four days in a week except for essential tasks (e.g., shopping for daily necessaries). Perceived overall compliance was satisfactory considering no lockdown has been implemented and some must maintain a normal working life. Keeping a physical distance of 1.5 meters, especially in public transportation, workspaces and restaurants, is demanding in crowded metropolitan districts such as Hong Kong. Flexible working schedules to avoid crowded transportation, home office working, and closure of unessential businesses may supplement these distancing measures [16].

Being female, older age, and having higher SES were associated with higher levels of perceived compliance with social distancing measures. Without the need for work-related essential tasks, the practice of stay-at-home and social distancing is easier for economically inactive people (e.g., students with class suspensions, homemakers, or retirees). The early reported higher mortality rate among older adults (aged 60+) [30] might have raised alarm, leading to higher compliance with home-confinement in the elderly. Females and people with higher SES were more vigilant in recognizing and practicing public health measures. One possible explanation is that women and people with a higher SES are more health conscious [31]. In contrast, males with lower SES may have more essential job requirements and less flexible working schedules, which may lead to lower compliance. Knowledge of infectious disease, social norms and perceived effectiveness of the measurements were reported to be decisive regarding level of compliance [32]. Timely epidemiological reports, combined with minute-by-minute case reports and open contact tracing, may have strengthened health communication and population-wide adoption of precautionary measures during this pandemic [3,33].

The perceived effectiveness of and compliance with social distancing were associated with lower risks of mental distress. Consistently, personal protection measures including mask wearing and household disinfection were associated with less stress and depressive symptoms, adjusted for social distancing. The association between social distancing and mental health remained robust after additional adjustment for personal protective measures. Perceived effectiveness and compliance with public health measures in preventing the infection for oneself and family members, and contributing to the safety and health of the community and country may strengthen feelings of security and alleviate mental distress related to the pandemic. A repeated cross-sectional study conducted in January, February and March 2020 has reported that perceived susceptibility to worries about being infected was decreasing, while confidence in protecting oneself was increasing in Hong Kong [2,3]. Personal protective measures such as mask wearing have become a social norm, and this perceived protection and solidarity may further enhance individual compliance [9]. Dynamic fluctuation in mental well-being is possible following different stages of the pandemic and the effectiveness of control measures. Areas experiencing severe outbreaks and with poor community control may encounter shortages in preventive supplies, more worries about being infected, less confidence in self-protection, less compliance with social directives and more serious mental health crises.

In contrast, staying at home for a longer time was associated with increased risks of depressive symptoms. Reverse causation is possible in which respondents with depressive symptoms might have tended to stayed-at-home. However, a crowded living environment, restricted face-to-face social interactions and restricted outside activities have led to a physically inactive and sedentary lifestyle for months, which may contribute to boredom, low mood, or mental distress. The elderly and less educated population might be more vulnerable regarding the processing of health information and sudden changes in social rules, especially in the digital world where public communication is mostly performed online [33,34]. We found in our subgroup analysis that older and less educated adults were more vulnerable to mental health symptoms if they stayed for more days at home. Increasing knowledge and promoting the use of preventive measures for the most vulnerable individuals is urgently needed in order to increase compliance with social distancing and maintain mental well-being. Health care providers have an important role in addressing mental distress as part of the pandemic response, improving compliance with social distancing and helping reduce the impact of COVID-19 on mental health. Decline in virus transmission and ease of public health restrictions are yet to come in this worsening pandemic. Social distancing is in need of collective effort for a long period of time in most affected countries and areas [18]. The long-term psychological impact in relation to the pandemic and social distancing measures requires further nation- and world-wide investigation.

### Limitations

Some limitations of the study should be noted. Causal relations could not be inferred in this cross-sectional study, where respondents with psychological distress were less likely to comply with public health measures. Pre-existing psychological problems were not collected, and residual confounders were not able to be excluded. Recall bias may exist in self-reporting measurements. The reasons for non-compliance with social distancing need further investigation for further public directives to be put into practice. The PSS-4 has a satisfactory construct validity [23] but a somewhat lower reliability (Cronbach’s α < 0.7). However, the reliability coefficient of 0.67 should not seriously attenuate validity [35] and the scale was consistent with PSS-14 and PSS-10 in correlations with other health-related variables, which provides a concurrent validity of the scale [24]. Simple measurements of mental health symptoms have restricted the clinical implications and more rigorous measurements are warranted. However, diagnostic instruments could be hard to implement in such a population-based survey. Respondents identified with higher stress level and having anxiety or depressive symptoms could benefit from further psychological assessment and support. We measured immediate mental health symptoms during the epidemic, and long term psychosocial responses such as social inactivity, post-traumatic stress disorder, and subthreshold or clinical anxiety and depression disorder will need further investigation.

## 5. Conclusions

Compliance with social distancing and stay-at-home is higher among female, older and educated respondents. Compliance with and perceived effectiveness of social distancing were associated with lower levels of stress, anxiety and depressive symptoms. More days stayed-at-home, however, may increase the risk of depressive symptoms, especially in older adults. Public health interventions are needed to protect the “new normal” in a future with or without COVID-19. The long-term psychological impact in relation to social distancing and stay-at-home requires further investigation. Social support and targeted interventions for the psychological well-being of the most underprivileged community members are urgently needed.

## Figures and Tables

**Table 1:** Sociodemographic characteristics by sampling method.

Sample Characteristic	Unweighted *n* (%) by Sampling Method			Combined (*N* = 1501)	
Sociodemographic and Mental Health Symptoms	Landline Telephone (*n* = 500)	Online Self-Administrated (*n* = 1001)	*p*-Value	Unweighted No. (%)	Weighted * (%)
Sex			0.08		
Male	208 (41.6)	464 (46.4)		672 (44.8)	47.5
Female	292 (58.4)	537 (53.6)		829 (55.2)	52.5
Age, years			0.11		
18–29	68 (13.6)	157 (15.7)		225 (15.0)	17.3
30–49	162 (32.4)	366 (36.6)		528 (35.2)	34.2
50–59	87 (17.4)	166 (16.6)		253 (16.9)	20.8
60–69	153 (30.6)	274 (27.4)		427 (28.4)	14.9
≥70	30 (6.0)	38 (3.8)		68 (4.5)	12.8
Marital status			0.06		
Never married	108 (21.6)	245 (24.5)		353 (23.5)	24.7
Married/Cohabitated	355 (71.0)	698 (69.7)		1053 (70.1)	66.1
Divorced/Separated	14 (2.8)	35 (3.5)		49 (3.3)	3.1
Widowed	23 (4.6)	23 (2.3)		46 (3.1)	6.1
Current living arrangement			0.83		
Living alone	21 (4.2)	49 (4.9)		70 (4.6)	5.4
Co-living with family members	471 (94.2)	936 (93.5)		1407 (93.7)	92.9
Co-living with other people	8 (1.6)	16 (1.6)		24 (1.6)	1.7
Educational attainment			0.64		
≤ Primary	88 (17.6)	159 (15.9)		247 (16.5)	23.2
Secondary	287 (57.4)	577 (57.6)		864 (57.5)	45.4
Tertiary	125 (25.0)	265 (26.5)		390 (26.0)	31.4
Employment status			0.86		
Full-time work	275 (55.0)	565 (56.4)		840 (55.9)	54.6
Part-time work	46 (9.2)	95 (9.5)		141 (9.4)	8.3
Student	19 (3.8)	45 (4.5)		64 (4.3)	4.8
Homemaker	71 (14.2)	131 (13.1)		202 (13.5)	11.1
Retired	68 (13.6)	118 (11.8)		186 (12.4)	17.2
Unemployed	21 (4.2)	47 (4.7)		68 (4.5)	3.9
Personal monthly income ^†^ (HK$)			0.62		
≤10,000	187 (37.4)	332 (33.2)		519 (34.6)	37.5
10,001–20,000	161 (32.2)	358 (35.8)		519 (34.6)	30.7
20,001–30,000	86 (17.2)	182 (18.2)		268 (17.9)	17.5
30,001–40,000	34 (6.8)	72 (7.2)		106 (7.1)	7.1
≥40,001	32 (6.4)	57 (5.7)		89 (5.9)	7.3
Perceived stress ^‡^ (Mean, SD)	7.13 (2.10)	7.27 (2.08)	0.98	7.23 (2.08)	7.20 (2.12)
Anxiety symptom ^§^	65 (13.0)	153 (15.3)	0.24	218 (14.52)	15.8
Depressive symptom ^¶^	60 (12.0)	146 (14.6)	0.17	206 (13.7)	14.8
Both anxiety and depression	42 (8.4)	94 (9.4)	0.16	136 (9.1)	10.4
Any anxiety or depression	83 (16.6)	205 (20.5)	0.07	288 (19.2)	20.1

Note: SD, Standard deviation; * Weighted by sex, age, educational attainment distributions of Hong Kong general population in 2016; ^†^ US $1 = HK $7.8; ^‡^ Measured by the Perceived Stress Scale-4 (range: 0–16), higher score indicating higher stress level. ^§^ Measured by the Generalized Anxiety Disorder-2 (range: 0–6), a score of ≥3 indicates anxiety symptom. ^¶^ Measured by the Patient Health Questionnaire-2 (range: 0–6), a score of ≥3 indicates depressive symptom.

**Table 2:** Prevalence of social distancing measures and compliance.

	**Prevalence**	
**Social Distancing Measures**	**Crude No. (%)**	**Weighted No. (%)**
Avoid going out	1107 (73.8)	1113 (74.2)
Avoid going to crowded places	1089 (72.6)	1091 (72.7)
Avoid social gathering of more than four people	878 (58.5)	897 (59.7)
Avoid going to high-risk places	860 (57.3)	822 (54.8)
Avoid handshaking, hugs and kisses	756 (50.4)	761 (50.7)
Keep 1.5 meters away from others in public places	614 (40.9)	644 (42.9)
Stay at home in past 7 days		
0 day	48 (3.2)	45 (3.0)
1 day	123 (8.2)	118 (7.9)
2 days	236 (15.7)	203 (13.5)
3 days	284 (18.9)	252 (16.8)
4 days	242 (16.1)	236 (15.7)
5 days	227 (15.1)	224 (14.9)
6 days	108 (7.2)	116 (7.7)
7 days	233 (15.5)	307 (20.5)
**Compliance with Social Distancing**	**Crude Mean (SD)**	**Weighted Mean (SD)**
Number of social distancing measures adopted *	3.53 (1.74)	3.55 (1.76)
Stay at home in past seven days	3.88 (1.97)	4.12 (2.05)
Perceived effectiveness of social distancing measures	7.36 (1.95)	7.34 (1.88)
Perceived compliance with social distancing measures	6.70 (1.93)	6.68 (1.98)

Note: SD, standard deviation; * Composite variable of total number of social distancing measures (range: 0–6).

**Table 3:** Social distancing measures and perceived compliance by sociodemographic factors.

		Number of the Social Distancing Measures Adopted ^¥^	Stay at Home in the Past Seven Days	Perceived Compliance with Social Distancing Measures
Sociodemographic		Adjusted β ^†^ (95%CI)	Adjusted β ^†^ (95%CI)	Adjusted β ^†^ (95%CI)
Sex	Male	-	-	-
	Female	0.09 (−0.09, 0.27)	0.49 (0.30, 0.68) ***	0.27 (0.07, 0.46) **
Age	18–29	-	-	-
	30–49	0.54 (0.23, 0.84) ***	0.53 (0.20, 0.87) **	0.31 (−0.02, 0.65)
	50–59	0.76 (0.40, 1.12) ***	0.45 (0.06, 0.85) *	0.49 (0.10, 0.89) *
	60–69	0.71 (0.34, 1.08) ***	0.29 (−0.11, 0.68)	0.88 (0.47, 1.29) ***
	≥70	1.48 (0.88, 2.07) ***	1.91 (1.18, 2.45) ***	1.45 (0.79, 2.11) ***
*p* for trend		<0.001	0.010	<0.001
Education	Primary or below	-	-	-
	Secondary	0.53 (0.25, 0.81) ***	0.09 (−0.20, 0.38)	0.29 (−0.02, 0.60)
	Tertiary	0.95 (0.59, 1.30) ***	0.80 (0.43, 1.17) ***	0.57 (0.18, 0.97) **
*p* for trend		<0.001	<0.001	0.004
Employment status	Full-time work	-	-	-
	Part-time work	0.00 (−0.33, 0.33)	0.86 (0.52, 1.21) ***	−0.04 (−0.41, 0.33)
	Student	−0.84 (−1.33, −0.35) ***	1.81 (1.30, 2.32) ***	0.34 (−0.20, 0.88)
	Homemaker	−0.21 (−0.54, 0.13)	1.80 (1.45, 2.15) ***	0.45 (0.08, 0.82) *
	Unemployed	0.08 (−0.37, 0.53)	2.07 (1.60, 2.53) ***	0.18 (−0.31, 0.68)
	Retired	−0.01 (−0.37, 0.35)	2.37 (2.00, 2.75) ***	0.65 (0.26, 1.05) ***
*p* for trend	-	0.44	<0.001	<0.001
Monthly income ^‡^ (HK$)	≤10,000	-	-	-
	10,001–20,000	−0.08 (−0.34, 0.19)	−0.51 (−0.79, −0.24) ***	0.33 (0.04, 0.62) *
	20,001–30,000	0.10 (−0.22, 0.42)	−0.38 (−0.70, −0.05) *	0.60 (0.25, 0.95) ***
	30,001–40,000	0.03 (−0.39, 0.44)	−0.17 (−0.60, 0.26)	0.73 (0.28, 1.19) **
	≥40,001	0.31 (−0.15, 0.77)	0.46 (−0.01, 0.94)	0.65 (0.14, 1.15) *
*p* for trend		0.16	0.085	<0.001

Note: SD, standard deviation; CI, confidence interval; * *p* < 0.05, ** *p* < 0.01, *** *p* < 0.001 ^¥^ Composite variable of total number of social distancing measures (range: 0–6); ^†^ Mutually adjusted for sociodemographic factors in the table; ^‡^ US $1 = HK $7.8.

**Table 4:** Association of mental health symptoms with social distancing measures.

Social Distancing Associated with Mental Health Symptoms	Stress (Range: 0–16)		Anxiety (Yes vs. No)		Depressive (Yes vs. No)	
	Model 1 Adjusted β (95%CI) *	Model 2 Adjusted β (95%CI) ^†^	Model 1 Adjusted β (95%CI) *	Model 2 Adjusted β (95%CI) ^†^	Model 1 Adjusted β (95%CI) *	Model 2 Adjusted β (95%CI) ^†^
Number of the social distancing measures adopted ^‡^	−0.11 (−0.17, −0.05) ***	−0.12 (−0.18, −0.06) ***	0.90 (0.82, 0.97) **	0.87 (0.79, 0.95) **	0.94 (0.86, 1.02)	0.92 (0.84, 1.00)
Stay at home in the past seven days	0.02 (−0.03, 0.08)	0.02 (−0.04, 0.07)	1.04 (0.96, 1.13)	1.04 (0.95, 1.13)	1.09 (1.00, 1.18) *	1.09 (1.00, 1.18) *
Perceived effectiveness of social distancing measures	−0.23 (−0.28, −0.17) ***	−0.19 (−0.24, −0.14) ***	0.79 (0.73, 0.85) ***	0.81 (0.75, 0.87) ***	0.79 (0.73, 0.86) ***	0.82 (0.75, 0.88) ***
Perceived compliance to social distancing measures	−0.26 (−0.31, −0.21) ***	−0.20 (−0.25, −0.14) ***	0.79 (0.73, 0.85) ***	0.82 (0.76, 0.89) ***	0.79 (0.74, 0.86) ***	0.84 (0.77, 0.91) ***

Note: CI, confidence interval; OR, odds ratio; * *p* < 0.05, ** *p* < 0.01, *** *p* < 0.001; ^*^ Model 1: Adjusted for sociodemographic factors including sex, age, educational attainment, employment status and monthly income; ^†^ Model 2: Additionally adjusted for the four personal protection measures; ^‡^ Composite variable of total number of social distancing measures (range: 0–6).

## Supplementary Materials

The following are available online at https://www.mdpi.com/1660-4601/17/18/6692/s1, Table S1: Association of Mental Health Symptoms with Personal Protection Measures; Table S2: Interaction of Mental Health Symptoms by Age and Education.
