# Supplementary material for: Social Distancing Compliance under COVID-19 Pandemic and Mental Health Impacts: A Population-Based Study
Source: Int J Environ Res Public Health. 2020 Sep 14;17(18):6692. doi: 10.3390/ijerph17186692 (PMC7560229; doi:10.3390/ijerph17186692)
Supplement: Supplementary file 1 [file ijerph-17-06692-s001.pdf]

Table S1. Association of mental health symptoms with personal protection measures.

	Prevalence	Stress	Anxiety	Depression
**Personal protection measures**	**Weighted n (%)**	**Adjusted β (95%CI)** [a]	**Adjusted OR (95%CI)** [a]	**Adjusted OR (95%CI)** [a]
Wear a surgical mask when going out	1398 (93.1)	−1.85 (−2.26, −1.44) ***	0.28 (0.17, 0.45) ***	0.30 (0.18, 0.49) ***
Wash hands with alcohol-based sanitizers	1328 (88.5)	−0.57 (−0.89, −0.24) ***	0.84 (0.54, 1.31)	0.61 (0.40, 1.05)
Use alcohol to clean daily necessities	1186 (79.0)	−0.49 (−0.75, −0.23) ***	0.61 (0.44, 0.86) **	0.47 (0.34, 0.66) ***
Add water to household drainage system	1144 (76.2)	−0.49 (−0.73, −0.24) ***	0.55 (0.40, 0.75) ***	0.46 (0.33, 0.63) ***

Note: CI, confidence interval; OR, odds ratio; * $p < 0.05$, ** $p < 0.01$, *** $p < 0.001$ [a] Adjusted for sociodemographic factors and total number of social distancing measures (range: 0–6).

Table S2. Interaction of mental health symptoms by age and education.

**Effect Modification of Age and Education**	**Adjusted β/OR (95% CI)** [a]		***p* for Interaction**	**Adjusted β/OR (95% CI)** [b]			***p* for Interaction**
	**Age Groups**			**Education Attainment**			
	**18–59 years**	**60+ years**		**Primary or below**	**Secondary**	**Tertiary**	
Stress level (β)							
Stay at home in past 7 days	0.00 (−0.08, 0.08)	0.04 (−0.04, 0.11)	0.55	0.11 (0.01, 0.22) *	0.02 (−0.05, 0.10)	−0.02 (−0.15, 0.10)	0.014
Perceived compliance with social distancing measures	−0.29 (−0.37, −0.22) ***	−0.27 (−0.34, −0.19) ***	0.34	−0.24 (−0.34, −0.14) ***	−0.30 (−0.37, −0.23) ***	−0.26 (−0.37, −0.14) ***	0.90
Anxiety (OR)							
Stay at home in past 7 days	0.98 (0.89, 1.09)	1.17 (1.05, 1.31) **	0.005	1.34 (1.07, 1.66) **	1.03 (0.93, 1.13)	1.01 (0.88, 1.17)	0.17
Perceived compliance with social distancing measures	0.79 (0.71, 0.87) ***	0.80 (0.72, 0.90) ***	0.42	0.81 (0.67, 0.98) *	0.78 (0.71, 0.87) ***	0.80 (0.69, 0.93) **	0.72
Depression (OR)							
Stay at home in past 7 days	1.03 (0.93, 1.13)	1.22 (1.08, 1.37) ***	0.008	1.46 (1.16, 1.85) ***	1.07 (0.97, 1.19)	1.00 (0.86, 1.16)	0.16
Perceived compliance with social distancing measures	0.77 (0.70, 0.86) ***	0.83 (0.74, 0.93) **	0.23	0.83 (0.68, 1.01)	0.78 (0.71, 0.86) ***	0.78 (0.68, 0.91) **	0.89

Note: CI, confidence interval; OR, odds ratio.; * $p < 0.05$, ** $p < 0.01$, *** $p < 0.001$; [a] Adjusting for sex, educational attainment and marital status; [b] Adjusting for sex, age and marital status.
